# Height-related risk factors for prostate cancer

**DOI:** 10.1054/bjoc.1999.0906

**Published:** 1999-12-08

**Authors:** A E Norrish, C U McRae, I M Holdaway, R T Jackson

**Affiliations:** Department of Community Health, University of Auckland, Auckland, New Zealand Departments of Urology Endocrinology, Auckland Hospital, Auckland, New Zealand

**Keywords:** prostate neoplasms, height, growth, familial, IGF-1

## Abstract

Previous studies have reported that adult height is positively associated with the risk of prostate cancer. The authors carried out a population-based case–control study involving 317 prostate cancer cases and 480 controls to further investigate the possibility that height is more strongly associated with advanced, compared with localized forms of this disease. Since the inherited endocrine factors, which in part determine height attained during the growing years, may influence the risk of familial prostate cancer later in life, the relationship with height was also investigated for familial versus sporadic prostate cancers. Adult height was not related to the risk of localized prostate cancer, but there was a moderate positive association between increasing height and the risk of advanced cancer (relative risk (RR) = 1.62; 95% confidence interval (CI) 0.97–2.73, upper versus lowest quartile, *P* -trend = 0.07). Height was more strongly associated with the risk of prostate cancer in men with a positive family history compared with those reporting a negative family history. The RR of advanced prostate cancer for men in the upper height quartile with a positive family history was 7.41 (95% CI 1.68–32.67, *P* -trend = 0.02) compared with a reference group comprised of men in the shortest height quartile with a negative family history. Serum insulin-like growth factor-1 levels did not correlate with height amongst men with familial or sporadic prostate cancers. These findings provide evidence for the existence of growth-related risk factors for prostate cancer, particularly for advanced and familial forms of this disease. The possible existence of inherited mechanisms affecting both somatic and tumour growth deserves further investigation. © 2000 Cancer Research Campaign


*British Journal of Cancer* (2000) **82**(1), 241–245
© 2000 Cancer Research Campaign
Article no. bjoc.1999.0906

### Height-related risk factors for prostate cancer

**AE Norrish[1], CU McRae[2], IM Holdaway[3] and RT Jackson[1]**

Department of [1]Community Health, University of Auckland, Auckland, New Zealand; Departments of [2]Urology and [3]Endocrinology, Auckland Hospital, Auckland, New Zealand.

**Summary** Previous studies have reported that adult height is positively associated with the risk of prostate cancer. The authors carried out a population-based case–control study involving 317 prostate cancer cases and 480 controls to further investigate the possibility that height is more strongly associated with advanced, compared with localized forms of this disease. Since the inherited endocrine factors, which in part determine height attained during the growing years, may influence the risk of familial prostate cancer later in life, the relationship with height was also investigated for familial versus sporadic prostate cancers. Adult height was not related to the risk of localized prostate cancer, but there was a moderate positive association between increasing height and the risk of advanced cancer (relative risk (RR) = 1.62; 95% confidence interval (CI) 0.97–2.73, upper versus lowest quartile, *P*-trend = 0.07). Height was more strongly associated with the risk of prostate cancer in men with a positive family history compared with those reporting a negative family history. The RR of advanced prostate cancer for men in the upper height quartile with a positive family history was 7.41 (95% CI 1.68–32.67, *P*-trend = 0.02) compared with a reference group comprised of men in the shortest height quartile with a negative family history. Serum insulin-like growth factor-1 levels did not correlate with height amongst men with familial or sporadic prostate cancers. These findings provide evidence for the existence of growth-related risk factors for prostate cancer, particularly for advanced and familial forms of this disease. The possible existence of inherited mechanisms affecting both somatic and tumour growth deserves further investigation. © 2000 Cancer Research Campaign

**Keywords**: prostate neoplasms; height; growth; familial; IGF-1

Several epidemiological studies have reported positive associations between prostate cancer risk and adult height (Le Marchand et al, 1994; Andersson et al, 1996, 1997; Giovannucci et al, 1997; Hebert et al, 1997). However, the risk for clinically important (advanced) prostate cancer has been examined separately by only one previous study, in which a stronger positive association with height was observed for advanced forms of this disease (Giovannucci et al, 1997). The significance of the association with height has not been determined, but it suggests the existence of important growth-related risk factors for prostate cancer, possibly involving endocrine mechanisms. For example, androgens and insulin-like growth factor-1 (IGF-1) may influence the attainment of height particularly during puberty (Keenan et al, 1993; Juul et al, 1995), and circulating levels of these hormones in adults have been positively associated with prostate cancer risk (Gann et al, 1996; Mantzoros et al, 1997; Chan et al, 1998). The underlying determinants of height are largely genetic. Familial risk factors for prostate cancer are also well-established with a two- to threefold increase in risk in men with one or more affected first-degree relatives (Giovannucci, 1995). The identification of susceptibility loci on chromosome 1 (Smith et al, 1996) and the X-chromosome (Xu et al, 1998) strongly supports the existence of inherited susceptibility genes for prostate cancer but the mechanisms involved in familial prostate cancer are unknown. In particular, a possible role played by growth-related endocrine mechanisms in familial prostate cancer has received little previous attention. The aims of our study were twofold; first, to investigate associations between adult height and the risk of localized compared with advanced prostate cancer, and secondly; to examine height-related risks for familial compared with sporadic prostate cancer.

*Received 9 March 1999*
*Revised 26 May 1999*
*Accepted 7 June 1999*

*Correspondence to:* AE Norrish

### MATERIALS AND METHODS

The Auckland Prostate Study is a population-based case–control study carried out in the greater metropolitan area of Auckland, New Zealand. The study population included all men aged 40–80 years normally resident in the Auckland area during the 13-month study recruitment period from January 1996. Since almost all men in this group with newly diagnosed prostate cancer attend urologists, urology clinics were used as the basis for case-attainment. In this study, all public hospital urology clinic attendees and patients attending five of seven private clinic urologists were eligible for participation (an estimated 91% of all eligible patients). Wherever possible, the study aimed to identify prostate cancer cases (aged 40–80 years) from a larger group of men who were recruited prospectively following referral to the urology clinics for investigation of prostate-related conditions. For these men, data collection preceded their knowledge of the prostate cancer diagnosis. This was designed to reduce recall bias and improve response rates. Histology reports were used as a back-up means of identifying and recruiting remaining prostate cancers from urology clinics retrospective to the date of diagnosis.

All cases were histologically confirmed, and patients with a previous diagnosis of prostate cancer were excluded. Sub-groups of men with ‘advanced’ and ‘localized’ prostate cancers were defined prior to study analyses. Advanced cancers comprised cases with either pathological or radiological (bone scintigraphy) evidence of spread beyond the prostate capsule or a combined

Gleason score greater than or equal to seven. Localized cancers included the mutually exclusive group with no evidence of extra-capsular extension and a combined Gleason score of six or less. Study controls comprised men aged 40–80 years, with no history of prostate cancer, randomly selected from the Auckland general electoral rolls. Control participants were group-matched to cases on a monthly basis during the study recruitment period using 10-year age groups and an approximate case:control ratio of 1:1.5. Approval to carry out the study was obtained from the Northern Regional Health Authority Ethics Committee and informed written consent was obtained from all participants.

Study participants completed self-administered questionnaires that collected personal, socio-demographic, anthropometric, dietary and lifestyle data. Height (without shoes) and weight (with light clothing) were self-reported. Participants were asked whether first-degree relatives (parents or siblings) had been diagnosed with cancer, and for each cancer, to specify the site and age at which it had first developed. Identical procedures were used for exposure data collection from cases and controls. Research nurses visited all participants at home to obtain blood samples and to check the completeness of responses to the questionnaires which had been posted to participants previously. Participants with missing responses were encouraged to fully complete the questionnaires (self-administered) at the time of the visit. Although the nurse-interviewers were not ‘blind’ to the case–control status of participants, neither they nor the participants were aware of specific hypotheses concerning prostate cancer risk.

During the study recruitment period, 10 ml of whole blood were collected in EDTA and transported promptly to the laboratory in chilled polystyrene containers. Serum samples were stored in cryovials (Greiner) at –82°C for an average period of 2 years prior to further analysis in October 1998, to determine serum IGF-1 levels. Serum IGF-1 was measured by radioimmunoassay after acid–ethanol cryoprecipitation, using a rabbit antiserum to recombinant human metIGF-1, and using metIGF-1 labelled with chloramine-T. The assay sensitivity was 2 $\mu g\ l^{-1}$ and the intra- and inter-assay coefficients of variation were 5% and 7% respectively (Gluckman et al, 1983). For the purposes of the current analyses, serum IGF-1 was measured for cases reporting a positive family history of prostate cancer (familial cases) and an age- and height-matched group of cases selected randomly from those with a negative family history (sporadic cases). For these participants, age was matched to within 1 year and height to within 5 cm. Prior to blood analyses it was estimated that a comparison involving 31 pairs would possess 80% power to detect a 20 $\mu g\ l^{-1}$ difference in mean serum IGF-1 between familial and sporadic cancer groups (assuming a variance of the differences of 40 $\mu g\ l^{-1}$ and alpha = 0.05).

Relative risks for total, localized and advanced prostate cancers were calculated for men in quartile categories for height, weight and body-mass index [weight (kg)/height $(m)^2$], with the reference group comprised of those in the lowest quartile (quartile categories were based upon distribution in the control group). The prostate cancer risk for men who reported a positive family history of at least one first-degree relative affected by prostate cancer was compared with those reporting a negative history. Men whose family histories of cancer were reported as ‘unknown’ were excluded from these analyses. Age-adjusted relative risks were calculated for prostate cancer, since controls were matched to cases by age group during recruitment. Multivariate relative risks were calculated using an unconditional logistic regression model. Age was included as a continuous variable in regression models, but other co-variates were included as categorical terms. A number of socio-demographic, anthropometric and dietary co-variates were considered as potential confounders of the association between family history, height and prostate cancer. Socio-economic status was defined by the participants’ usual current or former occupation (if retired), according to the modified Elley–Irving Classification (Johnston, 1983) which has been widely used in New Zealand population research. A test for overall trend across height categories used the *P*-values from a logistic regression model which included ordinal terms for each quartile of height, as well as confounding co-variates. The presence of an interaction between family history and height (continuous variable) was tested by including these two variables and a product term, along with other potentially confounding co-variates, in a logistic regression model.

### RESULTS

Participants in the Auckland Prostate Study included a total of 480 controls (71% response rate) and 317 prostate cancer cases (77% response rate), including 192 advanced cases and 125 localized cases. The socio-economic status of the control group was modestly elevated compared with cases, but due to the age-matched recruitment process there was little difference in age distribution.

There was a weak positive association between height and risk of total prostate cancer risk which was not statistically significant at the 95% level of confidence (Table 1). Although height was not associated with the risk of localized prostate cancer, the risk of advanced cancers, adjusted for age and socio-economic status, was more strongly associated with increasing height (relative risk, upper:lowest quartile = 1.62; 95% confidence interval (CI) 0.97–2.73, *P*-trend = 0.07). Adjustment for socio-economic status strengthened the positive associations with height since a weak positive association existed between socio-economic status and height in the study population. However, adjustment for a number of dietary co-variates made little difference to the observed relative risk (RR) estimates. Weight and body-mass index were not associated with prostate cancer risk (data not shown).

The RR for men with at least one first-degree relative reported to be affected by prostate cancer (compared with no affected first-degree relatives) was 2.84 (95% CI 1.46–5.50), based upon 26 cases and 15 controls with a positive family history. Table 2 presents height-related risks for prostate cancer, stratified by family history status. The positive association with height was stronger for men reporting a positive family history compared with those with a negative family history, although these analyses included relatively small numbers of men in the study with a positive family history. The multivariate relative risk of advanced prostate cancer for men in the upper height quartile with a positive family history was 7.41 (95% CI 1.68–32.67, *P*-trend = 0.02), compared with a reference group comprised of men in the shortest height quartile with a negative family history. A positive interaction between family history and height was observed for advanced prostate cancers (*P* = 0.04), but this was weaker for total cancers (*P* = 0.14).

In order to investigate the hypothesis that the difference in height-related risks observed between familial and sporadic

 © 2000 Cancer Research Campaign

**Table 1** Relative risk of prostate cancer and self-reported height, by quartile, for total, localized and advanced cancer cases, Auckland Prostate Study, 1996–1997

| | | Relative risk (95% CI), by height quartile[a] | | | | |
|---|---|---|---|---|---|---|
| | | Height quartile | | | | |
| | | **Q1 (< 170 cm)** | **Q2 (170–173 cm)** | **Q3 (173–179 cm)** | **Q4 (>179 cm)** | ***P* for trend** |
| Total cancers | Age-adjusted RR | 1.00 | 1.31 (0.86–2.00) | 1.31 (0.86–2.01) | 1.24 (0.81–1.91) | 0.39 |
| | Multivariate RR[b] | 1.00 | 1.34 (0.88–2.05) | 1.40 (0.91–2.16) | 1.39 (0.90–2.14) | 0.16 |
| | Number of cases:controls[c] | 58:110 | 88:129 | 85:120 | 82:121 | |
| Localized cancersd | Age-adjusted RR | 1.00 | 1.28 (0.73–2.27) | 1.10 (0.62–1.87) | 0.92 (0.51–1.68) | 0.68 |
| | Multivariate RR[b] | 1.00 | 1.11 (0.63–1.96) | 1.09 (0.61–1.95) | 1.00 (0.55–1.83) | 0.99 |
| | Number of cases:controls[c] | 26:110 | 37:129 | 33:120 | 28:121 | |
| Advanced cancerse | Age-adjusted RR | 1.00 | 1.37 (0.82–2.28) | 1.46 (0.88–2.44) | 1.50 (0.90–2.50) | 0.13 |
| | Multivariate RR[b] | 1.00 | 1.38 (0.83–2.31) | 1.55 (0.92–2.60) | 1.62 (0.97–2.73) | 0.07 |
| | Number of cases:controls[c] | 32:110 | 51:129 | 52:120 | 54:121 | |

[a] Numbers of controls in quartile categories varied due to rounding of self-reported heights and selection of cut-off values to the nearest cm. [b]Relative risk adjusted for age and socio-economic status in an unconditional logistic regression model. Reference group: men in lowest quartile. [c]Total numbers may be incomplete due to missing data for some observations. [d]Localized cancers defined as combined Gleason score ≤ six *and* no evidence of extra-capsular spread. [e]Advanced cancers defined as combined Gleason score ≥ seven *or* evidence of extra-capsular spread.

**Table 2** Height, family history and prostate cancer relative risk, for total and advanced cancer cases, Auckland Prostate Study, 1996–1997

| | | Relative risk (95% CI), by height quartile[a] | | | | |
|---|---|---|---|---|---|---|
| | | Height quartile | | | | |
| | | **Q1 (< 170 cm)** | **Q2 (170–173 cm)** | **Q3 (173–179 cm)** | **Q4 (>179 cm)** | ***P* for trend** |
| Total cancers: | | | | | | |
| Negative family history | Age-adjusted RR | 1.00[a] | 1.27 (0.81–1.99) | 1.15 (0.72–1.82) | 1.09 (0.69–1.74) | |
| | Multivariate RR[b] | 1.00[a] | 1.29 (0.82–2.03) | 1.22 (0.76–1.94) | 1.22 (0.76–1.96) | |
| | Number of cases:controls[c] | 49:96 | 75:118 | 67:112 | 65:112 | |
| Positive family history[d] | Age-adjusted RR | 1.30 (0.28–6.14) | 3.56 (0.99–12.76) | 4.02 (1.17–13.79) | 4.28 (1.05–17.36) | 0.02 |
| | Multivariate RR[b] | 1.27 (0.27–6.07) | 3.54 (0.97–12.97) | 4.78 (1.36–12.77) | 5.49 (1.31–22.94) | 0.004 |
| | Number of cases:controls[c] | 3:4 | 7:4 | 9:4 | 7:3 | |
| Advanced cancers: | | | | | | |
| Negative family history | Age-adjusted RR | 1.00[a] | 1.27 (0.74–2.19) | 1.18 (0.68–2.05) | 1.15 (0.66–1.99) | |
| | Multivariate RR[b] | 1.00[a] | 1.28 (0.74–2.20) | 1.25 (0.71–2.18) | 1.25 (0.71–2.20) | |
| | Number of cases:controls[c] | 29:96 | 45:118 | 41:112 | 40:112 | |
| Positive family history[d] | Age-adjusted RR | 0.78 (0.08–7.30) | 2.62 (0.55–12.41) | 2.17 (0.45–10.42) | 6.25 (1.46–26.71) | 0.06 |
| | Multivariate RR[b] | 0.81 (0.09–7.58) | 2.83 (0.58–13.73) | 2.72 (0.55–13.47) | 7.41 (1.68–32.67) | 0.02 |
| | Number of cases:controls[c] | 1:4 | 3:4 | 3:4 | 6:3 | |

[a] Reference group: men in the first quartile of height and no first degree relatives with prostate cancer. [b]Relative risk adjusted for age and socio-economic status in an unconditional logistic regression model. [c]Numbers are incomplete due to missing values for height and unknown family history for some observations. [d]≥ one first degree relative affected.

cancers may be attributable to differences in circulating levels of IGF-1, we compared serum levels of IGF-1 between groups. Mean serum IGF-1 levels were lower for familial prostate cancer cases compared with a random sample of age- and height-matched sporadic prostate cancers. There was no clear correlation between serum IGF-1 levels and increasing quartiles of height for either group (Table 3).

### DISCUSSION

This study confirms the findings of previous studies reporting progressive increase in prostate cancer risk associated with increasing adult height. Elevated height-related risks applied mainly to advanced cancers, and were greater for men reporting a positive family history of prostate cancer.

Incomplete response rates observed amongst cases (77%) and controls (71%) in our study are typical for population-based case–control studies and are unlikely to have resulted in selection biases in relation to height. The exclusion of cases attending two out of seven private urologists (an estimated 9% of the total number of cases) may have contributed to the observed imbalance in the socio-economic status of cases and controls but all analyses were adjusted for this variable. Previous validation studies have suggested that self-reported heights may be over-estimated by shorter men when compared with measured height (Palta et al, 1982; Millar, 1986; Stewart et al, 1987). However, such misclassification would be expected to lead to underestimation of the positive association observed between height and prostate cancer risk.

The relatively small numbers of men in our study with familial prostate cancer has resulted in limited precision of the estimates of

© 2000 Cancer Research Campaign 

**Table 3** Serum insulin-like growth factor-1 (IGF-1) in familial prostate cancer cases and an age- and height-matched sample of sporadic prostate cancers, by quartile of height, Auckland Prostate Study, 1996–1997

| | Serum IGF-1 (μg/l) – mean ± standard deviation | | | | |
|---|---|---|---|---|---|
| | | | Height quartile | | |
| | Q1 (< 170 cm) | Q2 (170 cm–173 cm) | Q3 (173–179 cm) | Q4 (>179 cm) | All heights |
| Familial cases ($n$ = 26) | 188 ± 68 | 152 ± 73 | 144 ± 49 | 160 ± 67 | 159 ± 61 |
| Sporadic cases ($n$ = 26) | 203 ± 114 | 189 ± 82 | 165 ± 67 | 173 ± 60 | 179 ± 72 |

effect derived from the analyses stratified by reported family history, and these findings will require confirmation by other studies. However, the magnitude of the increase in cancer risk observed for men in the highest quartile of height with a positive family history was relatively large. Furthermore, the progressive increase in risk across increasing height categories and the positive interaction observed between height and family history for advanced cancers strengthens the evidence for a true association.

The stronger association observed with advanced prostate cancers in our study suggests that height may act as a marker for an aetiological factor of relevance to clinically significant variants of prostate cancer (for example more aggressive clones of malignant cells) or possibly for a factor acting at a relatively late stage in the progression of localized to advanced disease. Only one previous observational study has examined risk in relation to prostate cancer stage, with very similar findings to our own (Giovannucci et al, 1997). A Swedish study which reported a stronger association between height and fatal compared with incident cases (Andersson et al, 1997) is also consistent with our findings.

Additional evidence for the existence of growth-related constitutional risk factors for prostate cancer is provided by the observation that prostate cancer risk is positively associated with birthweight (Tibblin et al, 1995; Ekbom et al, 1996). Our analyses further suggest that height-related risks may be greater for familial forms of this disease. A possible explanation is that in high-risk individuals, genetically-determined variability in circulating hormones and growth factors may influence both height attained during the growing years and familial prostate cancer risk later in life. Prostate epithelial cells are rich in IGF-1 receptors and the proliferative and apoptosis-inhibiting properties of IGF and binding proteins in prostate cell lines (Cohen et al, 1991, 1994; Rajah et al, 1997) suggest a potentially important role in cancer processes. An increased risk of prostate cancer has been positively associated with circulating IGF-1 levels in two previous studies (Mantzoros et al, 1997; Chan et al, 1998). Growth-related endocrine mechanisms for prostate carcinogenesis or cancer progression involving IGF-1 therefore seem plausible. Androgen-stimulated IGF-1 production has been shown to play a role in post-pubertal somatic growth (Keenan et al, 1993; Juul et al, 1995), although circulating IGF-1 has not been correlated with adult height amongst older men (Goodman-Gruen and Barrett-Connor, 1997; Chan et al, 1998). Our preliminary analyses provided no evidence to support the hypothesis that increased levels of circulating IGF-1 may explain the increased risk observed amongst taller men with familial prostate cancer, although the number of study participants involved in this analysis was relatively small. However, our observations did not allow the examination of effects of circulating levels of IGF-1 prior to diagnosis, or of local autocrine or paracrine production of IGF-1 in the prostate. Other possible links between somatic growth and tumour development might include local or systemic production of growth factors such as IGF-II or fibroblast growth factor, IGF binding proteins or growth factor receptors.

In summary, we have confirmed previous study findings indicating a positive association between adult height and the risk of prostate cancer, particularly advanced disease, and provide preliminary evidence for a greater height-related risk for familial prostate cancer. These findings suggest the existence of a factor or factors capable of inducing somatic growth and promoting the development of prostate carcinoma, a possibility deserving further study.

### ACKNOWLEDGEMENTS

This work was supported by the Cancer Society of New Zealand and the Health Research Council of New Zealand. The assistance of the following urologists with the recruitment of participants is acknowledged: Mr John Boulton, Mr Jon Cadwallader, Mr Roger Chambers, Mr Russell McIlroy, Mr Derek Rothwell and Mr Michael Rice. Special acknowledgement is made of technical assistance given by Ms Cherie Mulholland, Ms Susan Hawkins and Mrs Fleur O'Keefe and assistance with laboratory analyses provided by Ms Francis Murray, Auckland Hospital Endocrinology Laboratory.

### REFERENCES

Andersson S-O, Baron J, Bergstrom R, Lindgren C, Wolk A and Adami H-O (1996) Lifestyle factors and prostate cancer risk: a case–control study in Sweden. *Cancer Epidemiol Biomarkers Prev* **5**: 509–513

Andersson S-O, Wolk A, Bergstrom R, Adami H-O, Engholm G, Englund A and Nyren O (1997) Body size and prostate cancer: a 20-year follow-up study among 135 006 Swedish construction workers. *J Natl Cancer Inst* **89**: 385–389

Chan JM, Stampfer MJ, Giovannucci E, Gann PH, Ma J, Wilkinson P, Hennekens CH and Pollak M (1998) Plasma insulin-like growth factor-1 and prostate cancer risk: a prospective study. *Science* **279**: 563–566

Cohen P, Peehl DM, Lamson N and Rosenfield RG (1991) Insulin-like growth factors (IGFs), IGF receptors and IGF binding proteins in primary cultures of prostate epithelial cells. *J Clin Endocrinol Metab* **73**: 401–407

Cohen P, Peehl DM, Graves HCB and Rosenfield RG (1994) Biological effects of prostate specific antigen (PSA) as an IGF binding protein-3 (IGFBP-3) protease. *J Endocrinol* **142**: 407–415

Ekbom A, Hsieh CC, Lipworth L, Wolk A, Ponten J, Adami H-O and Trichopoulos D (1996) Perinatal characteristics in relation to incidence of and mortality from prostate cancer. *Br Med J* **313**: 337–341

 *© 2000 Cancer Research Campaign*

Gann PH, Hennekens CH, Ma J, Longcope C and Stampfer MJA (1996) A prospective study of sex hormone levels and risk of prostate cancer. *J Natl Cancer Inst* **88**: 1118–1126

Giovannucci E (1995) Epidemiological characteristics of prostate cancer. *Cancer* **75**: 1766–1777

Giovannucci E, Rimm EB, Stampfer MJ, Colditz GA and Willet WC (1997) Height, body weight and risk of prostate cancer. *Cancer Epidemiol Biomarkers Prev* **6**: 557–563

Gluckman PD, Johnson-Barret TJ, Butler J, Edgar BW and Gunn TR (1983) Studies of insulin-like growth factors-I and -II by specific radioligand assays in umbilical cord blood. *Clin Endocrinol* **19**: 405–413

Goodman-Gruen D and Barrett-Connor E (1997) Epidemiology of insulin-like growth factor-1 in men and women. *Am J Epidemiol* **145**: 970–976

Hebert PR, Ajani U, Cook NR, Lee I, Chan KS and Hennekens CH (1997) Adult height and incidence of cancer in male physicians (United States). *Cancer Causes Control* **8**: 591–597

Johnston R (1983) *A Revision of Socio-economic Indices for New Zealand*. New Zealand Council for Educational Research: Wellington

Juul A, Dalgaard P, Blum WF, Bang P, Hall K and Michaelson KF (1995) Serum levels of insulin-like growth factor (IGF)-binding protein-1 (IGFBP-3) in healthy infants, children, and adolescents: the relation to IGF-1, IGF-II, IGFBP-2, age, sex, body mass index, and pubertal maturation. *J Clin Endocrinol Metab* **80**: 2534–2542

Keenam BS, Richards GE, Ponder SW, Dakkas JS, Nagamani M and Smith ER (1993) Androgen-stimulated pubertal growth: the effects of testosterone and dihydrotestosterone on growth hormone and insulin-like growth factor-1 in the treatment of short stature and delayed puberty. *J Clin Endocrinol Metab* **76**: 996–1001

Le Marchand L, Kolonel LN, Wilkins LR, Myers BC and Hirohata T (1994) Animal fat consumption and prostate cancer: a prospective study in Hawaii. *Epidemiology* **5**: 276–282

Mantzoros CS, Tzonou A, Signorello LB, Tricopoulos D and Adami H-O (1997) Insulin-like growth factor-1 in relation to prostate cancer and benign prostatic hyperplasia. *Br J Cancer* **76**: 1115–1118

Millar WJ (1986) Distribution of body weight and height: comparison of estimates based upon self-reported and observed measures. *J Epidemiol Comm Hlth* **40**: 319–323

Palta M, Prineas RJ, Berman R and Hannan P (1982) Comparison of self-reported and measured height and weight. *Am J Epidemiol* **115**: 223–230

Rajah R, Valentinis B and Cohen P (1997) Insulin-like growth factor (IGF)-binding protein-3 induces apoptosis and mediates the effects of transforming growth factor-beta 1 on programmed cell death through a p53- and IGF-independent mechanism. *J Biol Chem* **272**: 12181–12188

Smith JR, Freije D, Carpten JD, Gronberg H, Xu J, Isaacs SD, et al (1996) A genome-wide search reveals a major susceptibility locus for prostate cancer on chromosome 1. *Science* **274**: 1371–1373

Stewart AW, Jackson RT, Ford MA and Beaglehole R (1987) Underestimation of relative weight by use of self-reported height and weight. *Am J Epidemiol* **125**: 122–126

Tiblin G, Eriksoon S, Cnattingius S and Ekbom A (1995) High birthweight as a predictor of prostate cancer risk. *Epidemiology* **6**: 423–424

Xu J, Myers D, Freije D, Isaacs S, Wiley K, Nusskern D, et al (1998) Evidence for a prostate cancer susceptibility locus on the X chromosome. *Nat Genet* **20**: 175–179

*© 2000 Cancer Research Campaign*
